# From Bench to Bedside: Personalized Genomics in the Diagnosis and Treatment of Osteomyelitis

**DOI:** 10.3390/antibiotics15020210

**Published:** 2026-02-14

**Authors:** Amir Human Hoveidaei, Arian Rahimzadeh, Sara Mohammadi, Pranav Thota, Kimia Vakili, Parsa Yazdanpanahi, Ali Homaei, Seyed Arad Mosalamiaghili, Jakob Adolf, Janet D. Conway

**Affiliations:** 1 International Center for Limb Lengthening, Rubin Institute for Advanced Orthopedics, Sinai Hospital of Baltimore, Baltimore, MD 21215, USA; jconway@lifebridgehealth.org; 2 Neuromusculoskeletal Research Center, Iran University of Medical Sciences, Tehran 14535, Iran; arianrahimzadeh1380@gmail.com (A.R.); saramohammadi89@gmail.com (S.M.); kimiavakili1998@gmail.com (K.V.); yazdanpanahiparsa@gmail.com (P.Y.); aradmosalami@gmail.com (S.A.M.); 3 School of Medicine and Health Sciences, George Washington University, Washington, DC 20052, USA; 4 Department of Surgery, Brigham and Women’s Hospital, Harvard Medical School, Boston, MA 02115, USA; ahomaei@bwh.harvard.edu; 5 Department of Paediatric Orthopaedics, Sofia Medical University, 1614 Sofia, Bulgaria; jakobadolf@pm.me

**Keywords:** host genetics, interleukin-1β, NLRP3 inflammasome, osteomyelitis, personalized medicine, single nucleotide polymorphism

## Abstract

Osteomyelitis (OM), an inflammatory condition of the bone tissue, is a complex orthopedic condition marked by chronic inflammation, diagnostic uncertainty, and recurrent infections. Despite standard treatments—including surgical debridement, antimicrobial therapy, and bone reconstruction—many patients continue to experience recurrence and treatment failure. Growing molecular evidence indicates that host genetic factors play a crucial role in shaping immune responses and influencing disease progression in OM. This narrative review synthesizes current knowledge from candidate gene single-nucleotide polymorphism (SNP) association studies to illustrate how specific genetic variations contribute to OM pathogenesis, diagnostic refinement, and treatment outcomes. We examined key immunogenetic variants within genes involved in inflammatory signaling, pathogen recognition, and neutrophil regulation. Our synthesis identifies a landscape of pro-inflammatory SNPs, such as IL-1β rs16944 and NLRP3 rs10754558, that are associated with increased susceptibility to chronic or post-traumatic OM, as well as SNPs that are associated with protective effects that may favor infection resolution, such as within the NOS2 and VDR genes. These SNP-driven differences in inflammasome activity, cytokine pathways, and oxidative stress responses highlight emerging opportunities for individualized therapeutic strategies. This review consolidates these variants, providing a genetic framework to analyze host susceptibility and differentiating high risk from protective genetic profiles. Integrating genomic insights into OM management represents a promising shift toward personalized medicine, enhancing diagnostic precision, informing targeted interventions, and improving prognostic assessment. Continued large-scale validation of candidate SNPs and translational genomic models will be essential to support their future clinical application.

## 1. Introduction

Osteomyelitis (OM) is a progressive and debilitating inflammation of bone tissue, primarily caused by infectious agents, most notably Staphylococcus aureus [1,2]. OM imposes a substantial burden on patients and healthcare systems due to its prolonged course, frequent relapses, and complex management requirements [3]. This burden is compounded by its substantial morbidity and significant mortality risk, with the incidence of OM-related deaths doubling between 1999 and 2020 and reaching 1.8 per 100,000 [4]. While the overall mortality risk is approximately 2%, this risk is elevated in specific patient populations, such as older patients and those with diabetes, as well as in high-risk subtypes and sequelae like vertebral osteomyelitis and osteomyelitis-associated septicemia [5]. Beyond mortality, the condition imposes profound morbidity, with chronic OM marked by persistent inflammation, bone necrosis, and recurrent infections that often result in permanent functional impairment [6]. These complications lead to an immense financial strain on healthcare systems; for instance, the 12-month treatment cost for complex OM cases has been estimated as high as $156,818 in a specific U.S. cohort [7]. OM is classified by etiology into several subtypes: Post-traumatic osteomyelitis (PTOM), hematogenous OM (HO), vascular OM, and chronic OM [8,9]. PTOM is commonly associated with trauma or surgical intervention, while hematogenous OM arises from the systemic dissemination of pathogens, predominantly in pediatric and immunocompromised individuals [8,10]. Vascular OM typically develops under conditions of impaired blood flow, such as diabetes mellitus, where insufficient perfusion facilitates microbial colonization. Chronic OM is marked by persistent inflammation and bone necrosis, often resulting from inadequately managed acute infections, and carries a high risk of recurrence [11]. Despite advancements in surgical techniques and antimicrobial therapy, the etiological determinants of OM remain incompletely understood [12]. Recent genomic research has shed light on the crucial role of single-nucleotide polymorphisms (SNPs) in modulating the host immune response to infection. The SNPs defined as single-base-pair variations in the DNA sequence can alter the function and expression of immune-related genes such as those encoding toll-like receptors (TLRs), cytokines, enzymes, antimicrobial peptides, and regulators of bone remodeling. These variations have been implicated in dysregulated inflammatory responses, increasing susceptibility to OM [1]. Recent advancements in genetic research have established a foundation for the potential integration of SNPs into clinical practice, suggesting that SNP profiling could eventually inform clinical decision-making and personalized treatment protocols for OM. While the clinical application of these markers remains in the investigational stage, SNP profiling offers a theoretical foundation for enhanced diagnostic precision and optimized individualized therapeutic protocols [1]. Understanding the genetic architecture of OM susceptibility presents a unique opportunity to deepen our insights into disease mechanisms and to develop individualized preventive and therapeutic strategies [13,14]. While previous reviews have successfully cataloged genetic determinants or provided broad narrative summaries of clinical associations, a critical translational gap persists. This review addresses this gap by explicitly integrating clinical case–control findings with functional laboratory evidence and subsequently discussing the potential for future clinical implementation. The aim of this study is to review current literature on specific SNPs, with an emphasis on the synthesis of mechanistic studies and clinical association data. Furthermore, this review explores an investigational framework of integrating genomic data with clinical findings that may, upon further validation, enhance early risk prediction, facilitate personalized treatment approaches, and improve long-term outcomes for patients suffering from this chronic and costly condition.

## 2. Role of Immunogenetic Single Nucleotide Polymorphisms (SNPs) in Osteomyelitis Pathogenesis

Evidence from candidate gene studies suggests an association between variations in immunomodulatory genes and altered host susceptibility to OM. Certain single-nucleotide polymorphisms (SNPs) have been linked to the amplification of inflammatory pathways and increased risk, while others are associated with protective trends or show no consistent association. The populations listed in Table 1 represent the current breadth of available peer-reviewed literature on this topic rather than a targeted selection of specific regions. Of note, most findings are population-specific and require validation in larger, multi-ethnic cohorts.

### 2.1. SNPs Associated with Increased Susceptibility to OM

#### 2.1.1. Interleukin-1α (IL-1α, rs1800587 −889 C/T)

Evidence for IL-1α involvement in osteomyelitis comes from a Spanish case–control study (Asensi et al., 2003), in which the TT genotype occurred in 25% of 52 OM patients compared with 8.3% of 109 controls (OR = 3.7, *p* = 0.0081) [15]. A Greek chronic OM cohort (Tsezou et al., 2008) supported this association, identifying the 889 C/T variant as a genetic risk factor [16]. However, a larger Chinese Han study (Jiang et al., 2020; 189 PTOM vs. 200 controls) found no significant association, pointing to population- or phenotype-specific heterogeneity [17]. Functional work indicates that the T allele enhances promoter activity and IL-1α expression, plausibly amplifying inflammatory responses and predisposing to chronic OM [18].

#### 2.1.2. Interleukin-1β (IL-1β, rs16944 −511 C/T; IL-1β rs1143634 +3953 C/T)

The promoter polymorphism rs16944 (−511 C/T) in the IL-1β gene has been investigated across multiple populations for its role in osteomyelitis susceptibility. In a Chinese Han case–control study by Yao et al. (2019), which included 155 OM patients and 134 controls, the CC genotype was associated with a significantly increased risk of disease (OR ≈ 1.8) [19]. Similar results were reported in a Greek case–control cohort by Tsezou et al. (2008), which examined patients with chronic OM and identified rs16944 as a genetic risk factor [16]. More recently, a Chinese Han study by Jiang et al. (2020) involving 189 post-traumatic OM cases and 200 controls confirmed that the C allele conferred elevated susceptibility to PTOM [17]. These findings are consistent with a systematic review by Xie et al. (2021), which highlighted rs16944 as one of the most frequently reported IL-1β variants associated with OM [14]. Furthermore, a narrative review by Zhou et al. (2024) underscored rs16944 as a candidate risk locus, while emphasizing that its impact appears to be population dependent, with some non-Asian cohorts failing to replicate the association [1]. The IL-1β rs1143634 (+3953 C/T) polymorphism has also been identified as a significant genetic marker for osteomyelitis. In a Spanish cohort, the TT genotype was significantly more frequent in OM patients compared to healthy controls (OR ≈ 5.1) [11]. Notably, this variant was found to be in linkage disequilibrium with the IL-1α rs1800587 −889 × T allele, complicating efforts to determine if its influence on OM risk is independent or a reflection of its association with the IL-1α gene. The accumulated evidence suggests that these IL-1β polymorphisms are associated with increased OM risk, potentially by modulating IL-1β expression and amplifying pro-inflammatory responses; however, genetic heterogeneity across populations warrants further large-scale studies.

#### 2.1.3. Interleukin-6 (IL-6, rs1800796 −572 G/C; rs1800795 −174 G/C)

Among IL-6 polymorphisms, the −572 G/C variant has demonstrated the most consistent association with osteomyelitis. In a case–control study from China by Jiang et al. (2020), which included 189 post-traumatic OM patients and 200 healthy controls, carriers of the C allele at rs1800796 showed a significantly increased risk across multiple genetic models (dominant OR ≈ 4.18) [17]. This finding is in line with a systematic review by Xie et al. (2021), which analyzed published data and concluded that the CC and CG genotypes at −572 G/C confer higher susceptibility to post-traumatic OM [14]. More recently, a narrative review by Zhou et al. (2024) reinforced these observations, emphasizing rs1800796 as a reproducible genetic risk factor for OM [1]. By contrast, evidence regarding the −174 G/C variant (rs1800795) has been inconsistent: in a Greek case–control study by Tsezou et al. (2008), involving chronic OM patients and matched controls, this SNP was associated with increased risk [16], whereas Jiang et al. (2020) reported no significant effect in their Chinese Han cohort [17]. Synthesizing these findings, the −572 G/C polymorphism emerges as a more consistent indicator of OM risk, in contrast to the −174 G/C variant, which appears to show significant geographic and ethnic variability.

#### 2.1.4. Interleukin-10 Promoter Variants (rs1800871 −819 C/T; rs1800872 −592 C/A; rs1800896 −1082 G/A)

The IL-10 promoter SNPs have been studied for their potential to modulate inflammatory resolution in osteomyelitis. In a Saudi case–control study by Osman et al. (2015) on hematogenous OM, which included patients with HO and healthy controls, the A allele at rs1800871 (−819 T/C) was significantly more frequent among cases, suggesting elevated susceptibility [20]. In contrast, a Chinese Han PTOM cohort (Jiang et al., 2020; 189 cases vs. 200 controls) found no significant associations for rs1800871, rs1800872, or rs1800896 with post-traumatic OM [17]. A narrative review by Zhou et al. (2024) further underscores that although IL-10 promoter variants are biologically plausible in reducing IL-10 expression and impeding inflammation resolution, genetic findings remain inconsistent among populations [1]. Overall, the reported associations point to IL-10 promoter variants as potential contributors to OM risk via downregulation of anti-inflammatory responses, though their precise role likely depends on ethnicity, genetic background, and OM subtype.

#### 2.1.5. Interleukin-10 Intronic Variants (rs3024491 and rs3024496)

Evidence for IL-10 intronic SNPs in osteomyelitis remains limited. In a Chinese Han case–control study by by Zhou et al. (2024) including 189 patients and 200 healthy controls, carriers of the rs3024491 T allele did not show a statistically significant increase in risk across standard genetic models, while rs3024496 demonstrated only a borderline/non-significant effect [1]. A focused systematic review (Xie et al., 2021) listed these variants among IL-10 polymorphisms evaluated in OM but did not identify them as robust risk markers [14]. Of note, a 2024 study in periprosthetic joint infection (PJI) reported allelic frequency differences for rs3024491 between infected and non-infected arthroplasty patients, suggesting potential relevance of IL-10 intronic variation to bone/joint infections more broadly [21]. However, this finding does not directly validate an association with OM. Overall, while intronic regulation of IL-10 (e.g., via mRNA stability/splicing) is biologically plausible, replication in independent, multi-ethnic OM cohorts is still lacking.

#### 2.1.6. Interleukin-4 Promoter Variants (rs2243250 −589 C/T; rs2243248 −1098 G/T)

Promoter variants in IL-4, such as rs2243250 (−589 C/T) and rs2243248 (−1098 G/T), have been evaluated for their role in osteomyelitis susceptibility. In a Greek case–control study by Tsezou et al. (2008) involving chronic OM patients and matched controls (81 patients vs. 110 controls), the T allele of rs2243250 was significantly more frequent among cases, with a weaker but similar trend for rs2243248 [16]. Conversely, a Chinese Han PTOM cohort by Jiang et al. (2020) (189 PTOM cases and 200 controls) found no significant associations for either SNP [17]. A systematic review by Xie et al. (2021) also listed these variants among IL-4 SNPs evaluated in OM but concluded that their association is less consistent across studies [14]. A recent narrative review by Zhou et al. (2024) reemphasizes this ethnic variability [1]. In summary, results suggest that IL-4 promoter variants may influence OM risk via modulation of macrophage polarization and possibly impaired bacterial clearance [22]. However, the evidence strength is moderate and likely population-specific.

#### 2.1.7. Interferon-γ (IFN-γ, rs2430561 +874 T/A)

The IFN-γ +874 T/A (rs2430561) variant has been studied for its effect on post-traumatic osteomyelitis risk. In a Chinese Han case–control study by Zhao et al. (2020), including 189 PTOM patients and 200 healthy controls, carriers of the A allele (in AT or AA genotypes) had significantly higher risk compared to TT homozygotes in both the dominant model (AA + AT vs. TT, OR = 1.82, *p* = 0.017) and heterozygous model (AT vs. TT, OR = 1.78, *p* = 0.029) [23]. The allele frequency of A was also elevated among cases (15.07%) versus controls (9.25%; OR = 1.742, *p* = 0.013). This association is echoed in the systematic review by Xie et al. (2021), which highlights rs2430561 as one of several SNPs with evidence of regulatory function and repeated association across studies. Functional data indicate that the +874 A allele is associated with lower IFN-γ expression, potentially compromising macrophage activation and cellular immunity. While replication outside of East-Asian cohorts remains limited, rs2430561 currently stands as a promising genetic marker for PTOM susceptibility [14].

#### 2.1.8. Natural Resistance-Associated Macrophage Protein 1/Solute Carrier Family 11 Member A1 (NRAMP1/SLC11A1) (rs17235409 1730 G/A)

NRAMP1 plays an essential role in intracellular pathogen control and has been investigated as a candidate susceptibility gene for osteomyelitis [24,25,26,27]. In a Chinese Han case–control study by Jiang et al. (2020), which included 189 PTOM patients and 200 healthy controls, carriers of the AG genotype at rs17235409 exhibited a significantly higher risk of osteomyelitis, accompanied by elevated inflammatory markers [17]. These findings were expanded in a larger Chinese Han cohort by Jiang et al. (2023) involving 336 PTOM cases and 368 controls, where the AG genotype was again significantly associated with increased susceptibility in both the dominant model (OR = 1.44, *p* = 0.037) and heterozygous model (OR = 1.45, *p* = 0.035) [28]. Moreover, AG carriers demonstrated higher levels of white blood cells and C-reactive protein, supporting a functional impact on systemic inflammation [28]. A narrative review by Zhou et al. (2024) reinforced these associations, while also emphasizing that validation outside of Asian populations is lacking [1]. Within Chinese Han populations, the NOS2 rs17235409 AG genotype has emerged as a reproducible genetic risk factor for PTOM, though replication in non-Asian cohorts remains necessary.

#### 2.1.9. BCL2-Associated X (BAX) (rs4645878 −248 G/A)

The BAX promoter polymorphism rs4645878 (−248 G/A) has been implicated in osteomyelitis susceptibility and apoptotic function. In a Spanish case–control study by Ocaña et al. (2007) involving 80 OM patients and 220 healthy controls, the A allele was significantly more frequent in patients (18.1%) than in controls (10.6%), with an OR = 1.81 [29]. The same study demonstrated lower rates of spontaneous apoptosis in peripheral neutrophils from A allele carriers and reduced Bax protein expression [29]. More recently, a narrative assessment by Zhou et al. (2024) included rs4645878 among SNPs examined in chronic osteomyelitis in a Spanish cohort, confirming that allele A remains over-represented and is considered a potential risk marker [1]. Functional evidence supports the view that the A allele decreases Bax expression, delays neutrophil apoptosis, and thereby sustains inflammation [14]. Although replication in non-European and non-Asian populations remains limited, the current body of evidence positions rs4645878 as a mechanistically plausible BAX variant associated with chronic and possibly post-traumatic OM risk.

#### 2.1.10. Interleukin-1 Receptor Antagonist (IL1RN, rs2234663 VNTR)

The IL1RN gene encodes the interleukin-1 receptor antagonist (IL-1Ra), a member of the IL-1 family that acts as a naturally occurring competitive inhibitor of IL-1α and IL-1β pro-inflammatory activities. By binding to the same receptors without initiating an inflammatory signal, IL-1Ra plays a critical role in determining the persistence or resolution of the inflammatory response [30]. In a Brazilian case–control study by De Souza et al. (2017) involving 39 OM patients and 114 controls, the IL1RN VNTR (rs2234663) *2/*2 genotype was associated with a significantly higher risk of developing OM (OR = 6.9) [31]. Furthermore, this specific genotype showed a significant association with Staphylococcus aureus infection, suggesting that carriers may possess a less effective host response to this common pathogen. Mechanistically, it is speculated that IL1RN *2 carriers have a lower IL-1Ra/IL-1β ratio, which would amplify pro-inflammatory activity and sustain acute inflammation rather than resolving it [31]. Collectively, the current body of evidence positions the IL1RN VNTR as a mechanistically plausible variant that influences the susceptibility to and severity of post-traumatic and chronic osteomyelitis.

### 2.2. SNPs with Protective Associations

#### 2.2.1. Interleukin-4 (IL-4, rs2070874 −34 C/T)

Evidence suggests a protective role for the T-carrying genotypes of rs2070874 in hematogenous osteomyelitis. In a Saudi case–control study by Osman et al. (2015) (King Fahad Medical City; HO patients vs. healthy controls), CT (vs. CC/TT) was protective, while the C-allele/CC increased risk (significant genotype and allele differences reported) [20]. This work used TaqMan genotyping across nine cytokine/cytokine-receptor SNPs and identified rs2070874 as one of the loci with opposite (protective) directionality for the T-carrying state. A systematic review (Xie et al., 2021) summarized available OM genetics and likewise noted rs2070874 (IL-4) among SNPs showing potential protective association, albeit with small study sizes and population variability [14]. A narrative review (Zhou et al., 2024) reiterated the protective effect of the heterozygous CT genotype at rs2070874 in the Saudi cohort and highlighted the need for multicenter validation [1]. Mechanistically, higher IL-4 activity could favor anti-inflammatory skewing and improved clearance dynamics [22].

#### 2.2.2. Natural Resistance-Associated Macrophage Protein 1/Solute Carrier Family 11 Member A1 (NRAMP1/SLC11A1) (rs3731865)

NRAMP1 regulates phagosomal metal transport and intracellular killing [27]. In a Chinese Han case–control study by Jiang et al. (2023) (PTOM; 336 cases vs. 368 controls), rs17235409 (risk) and rs3731865 were both tested. While rs17235409 showed significant risk in dominant/heterozygous models, rs3731865 exhibited a protective trend (dominant model OR = 0.67, *p* = 0.051; heterozygous OR = 0.69, *p* = 0.068), suggesting possible reduced susceptibility that narrowly missed conventional significance. The authors concluded that larger cohorts are needed to confirm a protective effect of rs3731865 [28]. A 2024 narrative review also cites these NRAMP1 findings and stresses replication in non-Asian populations [1].

#### 2.2.3. Interleukin-1 Receptor Antagonist (IL1RN, rs4251961 C/T)

In contrast to the risk-associated VNTR, other variants like IL1RN rs4251961 have demonstrated protective effects in different cohorts. In a Chinese Han study by Jiang et al. (2020) [17] involving 189 post-traumatic osteomyelitis (PTOM) patients and 200 healthy controls, the C allele of rs4251961 was identified as a protective factor. Specifically, carriers of the CT genotype demonstrated a significantly decreased risk of developing PTOM (OR: 0.409). While serological levels of IL-1Ra were not directly measured in this cohort, patients with the CT genotype exhibited lower relative serological levels of IL-6 and TNF-α compared to those with CC or TT genotypes. and exhibited lower relative serological levels of IL-6 and TNF-α.

### 2.3. SNPs with No Clear Association

#### Interleukin-2 (IL-2, rs2069762 −330 T/G)

Despite the immunological relevance of IL-2 and the prominence of its promoter variant rs2069762 (−330 T/G), no consistent genetic association with osteomyelitis has been demonstrated. A systematic review by Xie et al. (2021) included rs2069762 among many OM-related SNPs and found no significant effect [14]. To date, no primary human case–control study with sufficient sample size has confirmed an association between the G or T allele and OM risk. Interestingly, in an animal model of chronic osteomyelitis (Sprague-Dawley rats) by Mao et al. (2019), IL-2 expression was found to be significantly elevated during disease progression, and the STAT5/Treg pathway was hyperactivated; this highlights IL-2’s role in immune regulation of OM, but does not equate to evidence about the −330 T/G polymorphism [32]. While animal expression studies reinforce the biological involvement of IL2 in bone infection, current clinical data suggest that the rs2069762 polymorphism is not a reliable predictor of OM risk, highlighting the necessity for further genetic investigation in more diverse populations.

## 3. Receptor and Enzyme-Related Gene Variants in OM

**Figure 1:**
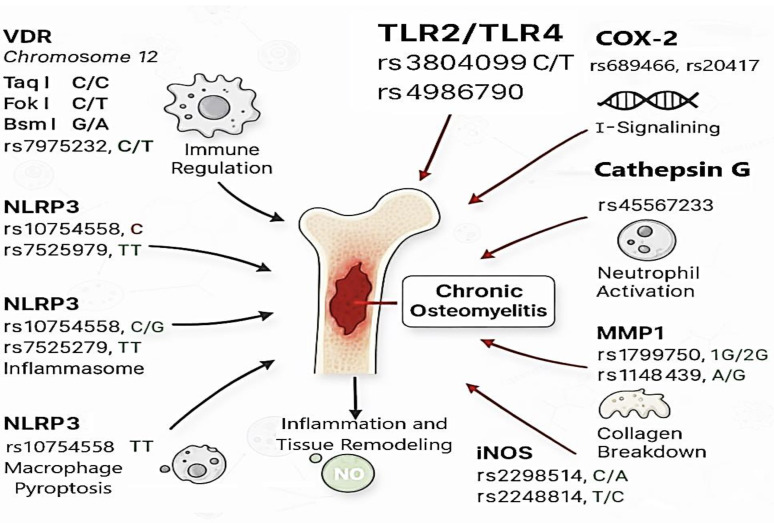
The figure illustrates single-nucleotide polymorphisms (SNPs) in genes involved in immune regulation, inflammation, and bone remodeling, including VDR, NLRP3, TLR2/4, COX-2, MMP1, and iNOS. Arrows indicate molecular interactions and biological processes such as cytokine signaling, macrophage pyroptosis, neutrophil activation, collagen breakdown, and tissue remodeling. These pathways are shown in the context of persistent osteomyelitis and the transition from acute to chronic states. Abbreviations: VDR, Vitamin D Receptor; NLRP3, NOD-like receptor family pyrin domain containing 3; TLR, Toll-like receptor; COX-2, Cyclooxygenase-2, Cathepsin G; MMP1, Matrix metalloproteinase-1; iNOS, Inducible nitric oxide synthase.

Genetic variation in receptor and enzyme-related genes has been implicated in susceptibility to osteomyelitis (OM) through its effects on immune recognition, inflammatory signaling, and tissue remodeling [14]. Depending on the specific locus and study population, single-nucleotide polymorphisms (SNPs) within these genes may function as risk alleles, confer protective effects, or show no consistent association, as illustrated in Figure 1 and summarized in Table 2.

### 3.1. SNPs Associated with Increased Susceptibility to OM

#### 3.1.1. Vitamin D Receptor (VDR)

The vitamin D receptor (VDR), located on chromosome 12, modulates innate and adaptive immunity and bone remodeling [33,34]. In a Chinese Han case–control study by Jiang et al. (2016) involving 233 patients with extremity chronic osteomyelitis and 200 healthy controls, carriers of the C allele at TaqI (rs731236) and FokI (rs2228570) had a significantly higher risk of OM [35]. These clinical findings seem to be consistent with biological models in which altered VDR signaling impairs antimicrobial macrophage functions [35]. By contrast, in a larger Chinese Han cohort by Zhao et al. (2022) (398 OM vs. 368 controls), ApaI (rs7975232) and BsmI (rs1544410) were associated with reduced OM susceptibility. In this same study, mechanistic experiments showed that VDR activation limits macrophage apoptosis by suppressing excessive ROS via VDR-Bmi1 signaling [36]. Taken together, current evidence supports a correlative link between VDR polymorphisms in OM risk, with TaqI/FokI associated with increased susceptibility in chronic OM (2016) and ApaI/BsmI showing protective associations in a larger cohort (2022). These divergent findings highlight population and SNP-specific heterogeneity between VDR and OM, underscoring the need for large-scale, multicenter trials to validate these markers for possible clinical use.

#### 3.1.2. Toll-like Receptors (TLR)

Toll-like receptors are key innate immune sensors, and their genetic variation has been implicated in osteomyelitis susceptibility [37]. In a Saudi Arabian case–control study by Osman et al. (2016) in patients with hematogenous OM, carriers of the C allele at TLR2 rs3804099 were significantly more frequent among cases, whereas the T allele and TT genotype were associated with protective trends, consistent with the hypothesized role of this SNP in modulating host–pathogen recognition [38]. For TLR4, two common variants, rs4986790 (Asp299Gly) and rs4986791 (Thr399Ile), have been associated with increased risk of osteomyelitis in European cohorts. In a Spanish case–control study by Montes et al. (2006) involving 235 OM patients and 361 controls, minor allele carriers (GG for rs4986790 and TT for rs4986791) had significantly higher susceptibility, consistent with altered TLR4 signaling and impaired host defense [39]. Functional studies further indicate that these polymorphisms modulate NF-κB/NFATc1 signaling, thereby promoting osteoclast differentiation and bone resorption. A recent narrative review by Zhou et al. (2024) reinforced these findings while emphasizing the need for replication in larger, multi-ethnic populations [1].

#### 3.1.3. NOD-like Receptor Family, Pyrin Domain Containing 3 (NLRP3)

NLRP3 encodes a cytosolic receptor that forms inflammasomes, promoting IL-1β maturation and pyroptosis in response to microbial or danger signals [40,41]. In a Chinese Han case–control study by Qu et al. (2024) involving 428 patients with chronic osteomyelitis and 368 healthy controls, the heterozygous CG genotype of rs10754558 was significantly associated with increased COM risk. While in the same cohort, the rs7525979 TT genotype showed a trend toward protection, although statistical significance was not reached [42]. A recent narrative review by Zhou et al. (2024) summarized current evidence indicating that NLRP3 polymorphisms may influence inflammasome activity, IL-1β production, and ultimately susceptibility to OM. The authors also emphasized the need for replication in larger cohorts, particularly in non-Asian populations [1].

#### 3.1.4. Cathepsin G (CTSG) (rs45567233, N125S)

In a Spanish case–control study by Pérez-Is et al. (2019), including 329 osteomyelitis patients and 415 healthy controls, the AG genotype at CTSG N125S (rs45567233) was significantly more frequent among cases compared to controls [43]. The G allele (AG + GG) was likewise more prevalent in patients. Functional analyses showed that G-allele carriers had higher CTSG enzymatic activity and lactoferrin levels compared with AA homozygotes. While these data suggest that the variant amplifies neutrophil-driven inflammatory responses, the exact mechanistic pathways through which this heightened activity drives OM susceptibility remain to be fully clarified [43]. A systematic review by Xie et al. (2021) incorporated these results, ranking rs45567233 among SNPs contributing to OM risk via enhanced CTSG and LF activity [14]. More recently, Zhou et al. (2024) highlighted the potential for CTSG N125S in OM susceptibility, while stressing that mechanistic pathways and replication across diverse populations remain to be clarified [1].

#### 3.1.5. Cyclooxygenase-2 (COX-2) (rs689466)

In a Chinese Han case–control study by Wang et al. (2017) involving 189 post-traumatic osteomyelitis (PTOM) patients and 220 healthy controls, the GG genotype at COX-2 rs689466 was significantly associated with increased PTOM risk under the recessive model [44]. Although allele-based and homozygous comparisons (GG vs. AA) did not reach statistical significance, the G allele showed a trend toward elevated susceptibility [44]. A recent narrative review by Zhou et al. (2024) reaffirmed rs689466 as a SNP associated with higher PTOM risk, while emphasizing that effect sizes and genetic models vary among studies [1]. Mechanistically, the rs689466 promoter variant has been shown to alter COX-2 (PTGS2) transcriptional activity, leading to increased prostaglandin synthesis and amplified inflammatory signaling. Such changes are biologically consistent with higher systemic inflammatory markers, including CRP and IL-6, although direct evidence confirming this specific transcriptional mechanism in osteomyelitis cohorts remains limited and requires further validation [45].

#### 3.1.6. Matrix Metalloproteinase-1 (MMP1) (rs1799750, rs1144393)

Matrix metalloproteinase-1 (MMP1), which degrades type I collagen and other extracellular matrix proteins, has been implicated in the pathogenesis of chronic osteomyelitis [46]. In a Chinese Han case–control study by Kong et al. (2017) evaluating rs1799750, the 2G allele was associated with increased susceptibility to osteomyelitis [47]. Similarly, rs1144393 G allele carriers were reported in multiple cohorts as having elevated risk, according to a systematic review by Xie et al. (2021) [14]. A recent narrative review by Zhou et al. (2024) confirms that patients with osteomyelitis show a higher incidence of the rs1144393 variant [1]. Collectively, these findings lead to the inference that the 2G and G alleles may upregulate MMP1 expression, potentially accelerating collagen degradation and tissue injury in OM. However, direct functional evidence confirming these SNP-driven expression levels in OM patients remains scarce, and reported effect sizes vary across populations.

#### 3.1.7. Tissue Plasminogen Activator (t-PA) (rs4646972)

In a Spanish case–control study by Valle-Garay et al. (2013) including 261 osteomyelitis patients and 299 healthy controls, homozygosity for the I allele (I/I genotype) of the t-PA Alu insertion/deletion polymorphism (rs4646972) was significantly more frequent among cases, and carrier status of the I allele was also elevated in patients [48]. Patients with/I genotype had significantly lower PAI-1/tPA complex levels compared to D allele carriers. Valle-Garay et al. suggest that the I/I genotype may increase susceptibility to bacterial osteomyelitis, possibly by promoting excess fibrinolysis and altered vascular remodeling [48]. A recent narrative review by Zhou et al. (2024) also includes rs4646972 among t-PA variants with replicated association to OM, though effect sizes and genotype frequencies vary by population [1].

**Table 2:** Key SNPs in receptor- and enzyme-related genes associated with osteomyelitis susceptibility, immune signaling, and tissue remodeling.

SNP	Association	Study	References
VDR (rs731236, rs2228570)	↑ Risk	Jiang et al., 2016—Chinese Han	[35]
VDR (rs7975232, rs1544410)	Protective	Zhao et al., 2022—Chinese Han	[36]
TLR2 (rs3804099)	Mixed	Osman et al., 2015—Saudi; Jiang et al., 2020—Chinese Han	[17,20]
TLR4 (rs4986790, rs4986791)	↑ Risk	European cohorts; Zhou et al., 2024	[1]
NLRP3 (rs10754558)	↑ Risk	Qu et al., 2024—Chinese Han	[42]
NLRP3 (rs7525979)	Protective	Qu et al., 2024—Chinese Han	[42]
CTSG (rs45567233, N125S)	↑ Risk	Pérez-Is et al., 2019—Spanish	[43]
COX-2 (rs689466)	↑ Risk	Wang et al., 2017—Chinese Han	[44]
COX-2 (rs20417)	No Association.	Multiple; Xie et al., 2021; Zhou et al., 2024	[1,14]
MMP1 (rs1799750, rs1144393)	↑ Risk	Kong et al., 2017—Chinese Han; reviews	[47]
t-PA (rs4646972, Alu I/D)	↑ Risk	Valle-Garay et al., 2013—Spanish	[48]
NOS2 (rs2297514)	Protective	Song et al., 2023—Chinese Han	[49]
NOS2 (rs2248814)	No Association.	Song et al., 2023—Chinese Han	[49]

Abbreviations: SNP, Single Nucleotide Polymorphism; VDR, Vitamin D Receptor; TLR, Toll-Like Receptor; NLRP3, NOD-Like Receptor Family Pyrin Domain Containing 3; CTSG, Cathepsin G; COX-2, Cyclooxygenase-2; MMP1, Matrix Metalloproteinase-1; t-PA, Tissue Plasminogen Activator; NOS2, Nitric Oxide Synthase 2.

### 3.2. SNPs with Protective Associations

Certain genetic variants appear to confer protection against osteomyelitis by enhancing immune regulation and limiting excessive inflammation [14]. In a Chinese Han study by Zhao et al. (2022) with 398 OM patients and 368 controls, the AA genotype at VDR rs7975232 (ApaI) was associated with reduced risk of fracture-related infections/OM, possibly through improved vitamin D signaling, antioxidant response, and preserved macrophage viability [36]. In the same population, Qu et al. (2024) studied NLRP3 rs7525979, finding the TT genotype associated with lower susceptibility to PTOM in homozygous and recessive genetic models [42]. Likewise, in a case–control study by Song et al. (2023) (336 PTOM vs. 368 controls), NOS2 rs2297514 genotype CC showed significantly decreased risk (recessive and homozygous models), with lower inflammatory biomarkers (especially CRP) among CC carriers compared to TT [49]. Collectively, these findings indicate that protective SNPs may function through multiple mechanisms, including enhanced anti-inflammatory capacity, stronger antioxidant defenses, and suppression of inflammasome or oxidative stress pathways.

### 3.3. SNPs with No Clear Association

#### 3.3.1. Cyclooxygenase-2 (COX-2) (rs20417)

In contrast to COX-2 rs689466, which has shown an association with PTOM risk, the rs20417 promoter polymorphism has not demonstrated a consistent link to osteomyelitis [44]. Existing case–control studies have failed to identify significant differences in genotype or allele frequency between OM patients and healthy controls. Both a systematic review by Xie et al. (2021) and a narrative synthesis by Zhou et al. (2024) conclude that the current evidence for rs20417 is inconclusive, underscoring the need for further investigation in larger, multi-ethnic cohorts [1,14].

#### 3.3.2. Nitric Oxide Synthase 2 (NOS2) (rs2248814)

Similarly, the NOS2 rs2248814 polymorphism has not been associated with OM risk in available studies. In the Chinese Han PTOM cohort by Song et al. (2023) (336 cases vs. 368 controls), no significant genotypic or allelic associations were detected between patients and controls [49]. These findings are consistent with systematic and narrative reviews by Xie et al. (2021) and Zhou et al. (2024), both of which conclude that rs2248814 is unlikely to contribute to OM susceptibility [1,14]. Nevertheless, given the central role of NOS2 in nitric oxide production and inflammatory regulation, a functional contribution remains biologically plausible and warrants further investigation [50].

## 4. Personalized Genomics in Osteomyelitis Treatment and Prognosis

**Figure 2:**
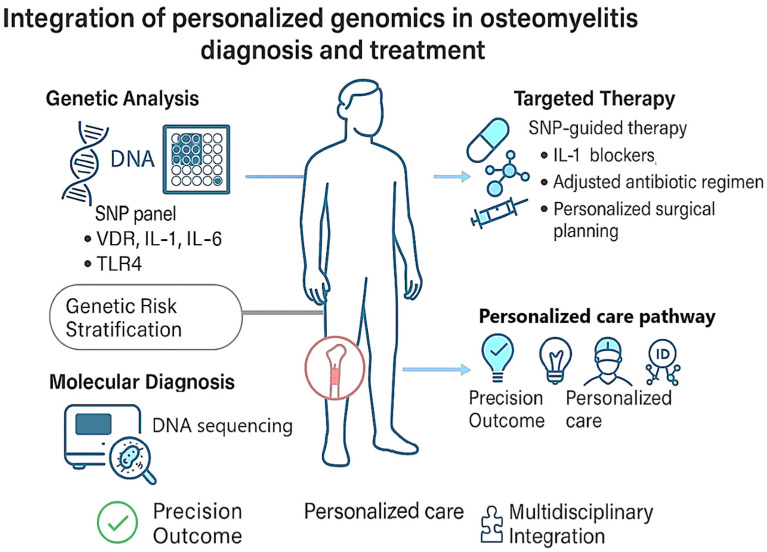
The schematic illustrates a conceptual framework for integrating immunogenetics into osteomyelitis management, depicting a hypothetical workflow from genetic profiling and molecular diagnostics to potential clinical applications. SNP profiling of key genes (VDR, IL-1, IL-6, TLR4) may support genetic risk stratification and molecular diagnosis. These insights could inform future targeted therapeutic strategies, optimized antibiotic regimens, and personalized surgical planning, contributing to precision medicine and multidisciplinary care. These approaches are conceptual and require further validation before routine clinical implementation. Abbreviations: SNP, single-nucleotide polymorphism; VDR, Vitamin D Receptor; IL, Interleukin; TLR4, Toll-like receptor 4.

Personalized genomics is emerging as a powerful tool in understanding and managing osteomyelitis (OM) (Figure 2). Genetic variations, particularly single-nucleotide polymorphisms (SNPs), have been shown to significantly influence individual susceptibility to infection, immune response, and treatment outcomes. For example, systematic reviews [1] summarize associations between SNPs in VDR, IFN-γ, TLRs, and interleukins and variable OM risk across populations, highlighting the potential for risk stratification and targeted intervention. Genome-wide association studies (GWAS) and large case–control cohorts [17,49,51] offer valuable insights for identifying high-risk individuals and enabling precision diagnostics. Moreover, advances in molecular techniques like metagenomic sequencing [52,53] enhance early pathogen detection and resistance profiling, further supporting individualized treatment strategies, though continued research is needed to translate these insights into standardized care. The current therapeutic strategies for OM consist of a multifaceted approach including radical surgical debridement, systemic and localized antibiotic administration, structural reconstruction of affected bone and soft tissues, and tailored rehabilitation programs. Treatment decisions must be guided by several factors such as anatomical location and chronicity of infection, pathogen type and virulence, host immune competence, and patient-specific considerations. As such, multidisciplinary involvement is essential [54]. The incorporation of surgeons, infectious disease experts, and clinical pharmacists enhances therapeutic outcomes. Given the growing recognition of host genetics in infection response, the inclusion of geneticists in clinical decision-making teams is increasingly warranted. Within an investigational framework, SNP-guided therapies may theoretically offer the potential for individualized treatment strategies based on a patient’s unique genetic architecture. Although clinical trials in OM remain limited, preliminary findings are promising. For instance, polymorphisms in NLRP3, a gene central to inflammasome activation, are associated with heightened inflammatory responses [42]. In patients harboring such SNPs, the use of experimental NLRP3 inhibitors, which are not currently approved for clinical OM management, has been proposed to attenuate inflammasome-mediated tissue damage and improve recovery. In parallel, a large prospective cohort study by McNally et al. [55] including 100 chronic PTOM patients tested local antibiotic carriers (calcium sulphate–tobramycin composite) and reported 94% infection eradication at final follow-up, underscoring the clinical benefit of integrating tailored antimicrobials into OM management. Additionally, targeting pro-inflammatory mediators like interleukins (e.g., IL-1, IL-6) or pathways linked to oxidative stress could theoretically provide a therapeutic advantage in genetically predisposed individuals. This targeted approach not only enhances efficacy but also minimizes systemic side effects, thereby optimizing patient-specific care [14]. As knowledge of host–pathogen–genome interactions deepens, the design of custom therapeutics based on SNP profiles has the potential to become a cornerstone in the management of OM. In regard to prognosis, OM, particularly in its chronic form, is associated with a high risk of recurrence and incomplete eradication despite aggressive treatment. Recent evidence underscores the pivotal role of host genetic factors in influencing disease persistence [1]. Polymorphisms in immune-regulatory genes such as NLRP3, IL-1β, IL-6, and TNF-α have been implicated in poor prognosis due to their roles in sustaining chronic inflammation and impaired resolution of infection. For example, in a large case–control study by Qu et al. [42]. involving 428 chronic OM patients and 368 controls (Chinese Han), carriers of the CG genotype at NLRP3 rs10754558 showed a ~60% higher risk of chronic OM compared to GG homozygotes, suggesting persistent inflammasome activation and relapse risk. Conversely, certain SNPs, such as the NOS2 rs2297514 CC genotype, have been associated with a more favorable outcome. In the context of treatment and prognosis, the identification of high-risk genetic profiles represents a proposed framework to inform more accurate risk stratifications, closer monitoring, and the use of adjunctive or extended therapies to prevent recurrence. Conversely, recognizing protective genotypes may help tailor treatment duration and reduce overtreatment. In conclusion, response to treatment and prognosis in OM seems to be a multifactorial outcome influenced not only by microbial and clinical parameters but also by the patient’s genetic landscape. Future research should aim to validate specific SNPs as biomarkers of prognosis, potentially allowing for their eventual integration into clinical decision-making algorithms to improve long-term outcomes and quality of life in affected patients.

## 5. Conclusions

In summary, the development and progression of osteomyelitis (OM) are significantly influenced by a complex interplay of host immunogenetic factors. This review synthesized current evidence demonstrating that specific SNPs in genes such as VDR (rs731236, rs2228570), TLR2 (rs3804099), NLRP3 (rs10754558), and CTSG (rs45567233) play roles in modulating the inflammatory response in OM. By integrating clinical association data with functional mechanistic studies, this review delineates a putative pathogenic landscape of pro-inflammatory and protective single-nucleotide polymorphisms (SNPs). While the clinical application of these markers remains in the investigational stage, integrating genomic insights into osteomyelitis management offers a potential framework for precision orthopedics, highlighting future pathways for more tailored diagnostic and therapeutic strategies. Future research should prioritize large-scale validation of candidate SNPs and the development of translational genomic models, thereby bridging genomic discoveries to bedside clinical practice.

## Figures and Tables

**Table 1:** Summary of SNPs in key immune-related genes and their reported impact on osteomyelitis.

SNP	Category	Population
IL-1α rs1800587	↑ Susceptibility	Spain, Greece; null in China
IL-1β rs16944	↑ Susceptibility	China, Greece
IL-1β rs1143634 (+3953 C/T)	↑ Susceptibility	Spain
IL-6 rs1800796	↑ Susceptibility	China
IL-6 rs1800795	↑ Susceptibility	Greece, China
IL-10 promoter (−819, −592, −1082)	↑ Susceptibility	Saudi, China
IL-10 intronic (rs3024491/96)	↑ Susceptibility	China; PJI data
IL-4 promoter (−589, −1098)	↑ Susceptibility	Greece, China
IFN-γ rs2430561	↑ Susceptibility	China
NRAMP1 rs17235409	↑ Susceptibility	China
BAX rs4645878	↑ Susceptibility	Spain
IL-1 RN rs2234663	↑ Susceptibility	Brazil
IL-4 rs2 070874	Protective	Saudi Arabia
IL-1 RN rs4251961	Protective	China
NRAMP1 rs3731865	Protective	China
IL-2 rs2069762	No Association	Reviews: animal data

Abbreviations: SNP, Single Nucleotide Polymorphism; IL, Interleukin; IFN-γ, Interferon gamma; NRAMP1, Natural Resistance-Associated Macrophage Protein 1 (also known as SLC11A1); BAX, Bcl–2–associated X protein.

## Data Availability

No new data were created or analyzed in this study. Data sharing is not applicable to this article.

## Abbreviations

The following abbreviations are used in this manuscript:

BAX	BCL2-Associated X
COX-2	Cyclooxygenase-2
CTSG	Cathepsin G
GWAS	Genome-wide association study
IFN-γ	Interferon-gamma
IL	Interleukin
IL-1β	Interleukin-1 beta
iNOS	Inducible nitric oxide synthase
MMP1	Matrix metalloproteinase-1
NLRP3	NOD-like receptor family pyrin domain-containing 3
NOS2	Nitric Oxide Synthase 2
OM	Osteomyelitis
PTOM	Post-traumatic osteomyelitis
SNP	Single-nucleotide polymorphism
t-PA	Tissue plasminogen activator
TLR	Toll-like receptor
VDR	Vitamin D receptor
